# Research trends in estrogen and rheumatoid arthritis: a systematic bibliometric approach

**DOI:** 10.1186/s13293-025-00806-4

**Published:** 2025-12-12

**Authors:** Xiaoling Dai, Pan Li, Di Pan

**Affiliations:** 1 Shanghai Putuo Traditional Chinese Medicine Hospital, Shanghai, China; 2 Longhua Hospital, Shanghai University of Traditional Chinese Medicine, Shanghai, China; 3 Shanghai General Hospital, Shanghai Jiao Tong University School of Medicine, Shanghai, China

**Keywords:** Rheumatoid arthritis, Estrogen, Bibliometrics, Cytokines, Inflammation

## Abstract

**Background:**

Rheumatoid arthritis (RA) is a chronic autoimmune disease. An increasing body of evidence indicates that hormones, particularly estrogen, play a significant role in RA. To date, no bibliometric studies have been conducted specifically on the role of estrogen in RA. This study seeks to perform a bibliometric analysis to elucidate research trends concerning estrogen in the context of RA from a comprehensive and systematic viewpoint.

**Methods:**

We extracted literature pertaining to estrogen in RA from the Web of Science Core Collection (WOSCC) database, up to April 22, 2025. Research trends in this domain were analyzed utilizing bibliometric software tools, VOSviewer and Bibliometricx.

**Results:**

A total of 1,009 literatures were included in this study. Articles in this field were first published in 1951 and have shown an overall upward trend since 1982. The United States and China were the countries that contributed the most articles, while the University of Genoa was the most contributing affiliation. CUTOLO M is the most prolific and most cited author. Arthritis and Rheumatology is the most published and cited journal in this field in the world. The primary focus of research in this area encompasses the evidence, mechanisms, and practical applications of estrogen’s involvement in RA. In addition, the key words such as “cytokines”, “inflammation”, “immune response”, “oxidative stress”, and “endometriosis” appear most frequently, which indicates that the mechanism research of estrogen’s participation in RA has been a research hotspot in recent years.

**Conclusion:**

This study reflects the degree of academic interest in the potential link between estrogen and RA. This also lays a foundation for continuous research in this field and provides certain insights for future research directions.

## Abstract (plain language summary)

Rheumatoid arthritis (RA) is a chronic autoimmune disease. More and more evidence indicates that estrogen plays an important role in rheumatoid arthritis. This is the first bibliometric study on estrogen and RA. This study aims to clarify the research trends of estrogen in rheumatoid arthritis from a comprehensive and systematic perspective through bibliometric analysis. We found that articles in this field were first published in 1951 and have generally shown an upward trend since 1982. The United States and China are the countries that have contributed the most articles, and the University of Genoa is the institution that has contributed the most. CUTOLO M is the most prolific and frequently cited author. Arthritis & Rheumatology is the most published and cited journal in the world in this field. The main research focuses in this field include the evidence, mechanism and practical application of estrogen in rheumatoid arthritis. In addition, keywords such as “cytokines”, “inflammation”, “immune response”, “oxidative stress”, and “endometriosis” appear most frequently, indicating that the research on the mechanism of estrogen’s involvement in RA has been a hot topic in recent years. This study reflects the degree of academic interest in the potential link between estrogen and RA. This also lays a foundation for continuous research in this field and provides certain insights for future research directions.

## Highlights

1. This is the first bibliometric study on estrogen and RA.
2. This study reflects the degree of academic interest in the potential link between estrogen and RA.
3. The primary focus of research in this area encompasses the evidence, mechanisms, and practical applications of estrogen’s involvement in RA.
4. The key words such as “cytokines”, “inflammation”, “immune response”, “oxidative stress”, and “endometriosis” appear most frequently, which indicates that the mechanism research of estrogen’s participation in RA has been a research hotspot in recent years.

## Introduction

Rheumatoid arthritis (RA) is a chronic systemic disorder characterized by articular involvement and a range of extra-articular manifestations [1]. Its pathogenesis is highly complex, involving both genetic and environmental factors [2]. The global incidence rate of RA is approximately 0.5%, with women exhibiting an incidence rate 2 to 3 times higher than that of men [3]. For different genders, sex hormones play a crucial role in it. Among them, estrogen is the female sex hormone that has been most extensively studied for its role in regulating the pathogenesis of RA [4]. It is reported that estrogen can affect the occurrence and development of RA [5, 6].

After numerous studies on estrogen and RA have been conducted, a systematic and comprehensive literature review is helpful for understanding the current research status and guiding future research directions. Bibliometric analysis refers to the quantitative analysis of knowledge in a specific discipline by using mathematical and statistical methods [7, 8]. It can effectively assess the contributions of journals, affiliations, and countries within a specific research domain, thereby aiding in the prediction of developmental trends in that field [9]. Integrating bibliometric analysis offers insights into the evolving research on estrogen’s role in RA. By systematically reviewing literature, we aim to highlight key contributions and suggest future research paths. This method enhances understanding of research findings and collaborations shaping current knowledge. Furthermore, bibliometrics has not yet emerged in the fields of estrogen in RA. This study seeks to elucidate the current research landscape and emerging trends concerning the role of estrogen in RA through a bibliometric analysis. By systematically reviewing relevant literature, this study evaluated the research progress, identified the main key issues, and explored the potential future directions in this field, thereby providing certain insights for clinical practice and subsequent research work.

## Materials and methods

### Data collection

**Fig. 1:**
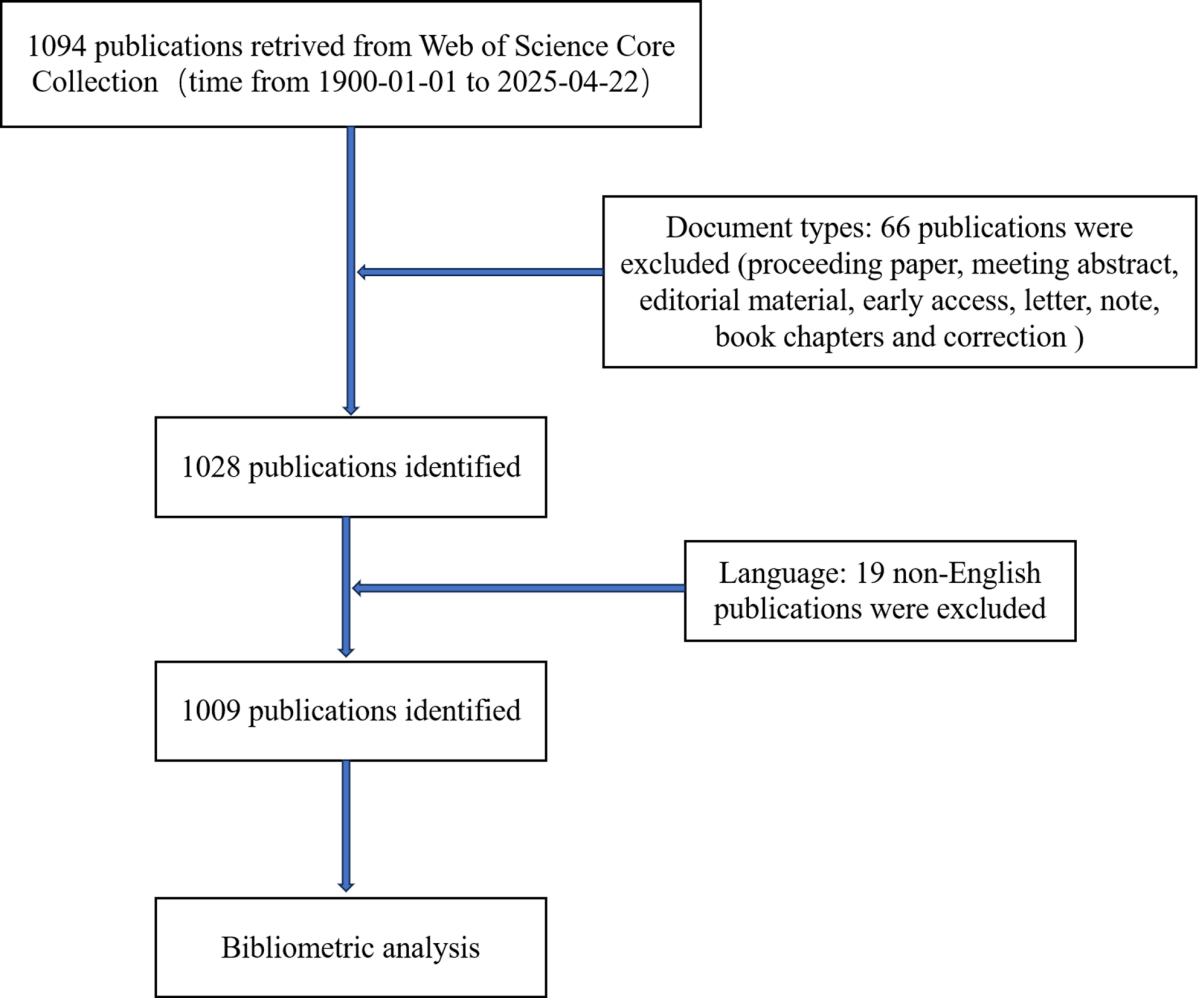
Detailed flowchart of retrieval

The Web of Science Core Collection (WOSCC) was selected for this analysis due to its status as one of the most comprehensive, systematic, and authoritative databases, encompassing internationally recognized and influential academic journals, and its widespread use in bibliometric studies [10]. The literature search, completed on April 22, 2025, ensures up-to-date results. We conducted a literature search using the following keywords: Topic = (“estrogen”) and Topic = (“rheumatoid arthritis”). Select the document types of English articles and reviews. Finally, 1,009 literatures were obtained. The inclusion and exclusion criteria are shown in Fig. 1.

### Data analysis

Bibliometric analysis and visualization were performed using Vosviewer version 1.6.20 [11] and Bibliometricx [12]. VOSviewer can be used to represent the cooperative relationships among countries/regions or affiliations, as well as the keyword co-occurrence network. Bibliometricx facilitates the understanding of publication and collaboration patterns across countries/regions and affiliations, author productivity timeline, article citations, journal publication timeline, topic evolution, and thematic map. These tools enable us to present basic information about countries, affiliations, authors, journals, keyword co-occurrence, and references in the research field of estrogen and RA intuitively and clearly, thereby helping to understand the research trends in this field.

## Results

### Analysis of publication output and trends

**Fig. 2:**
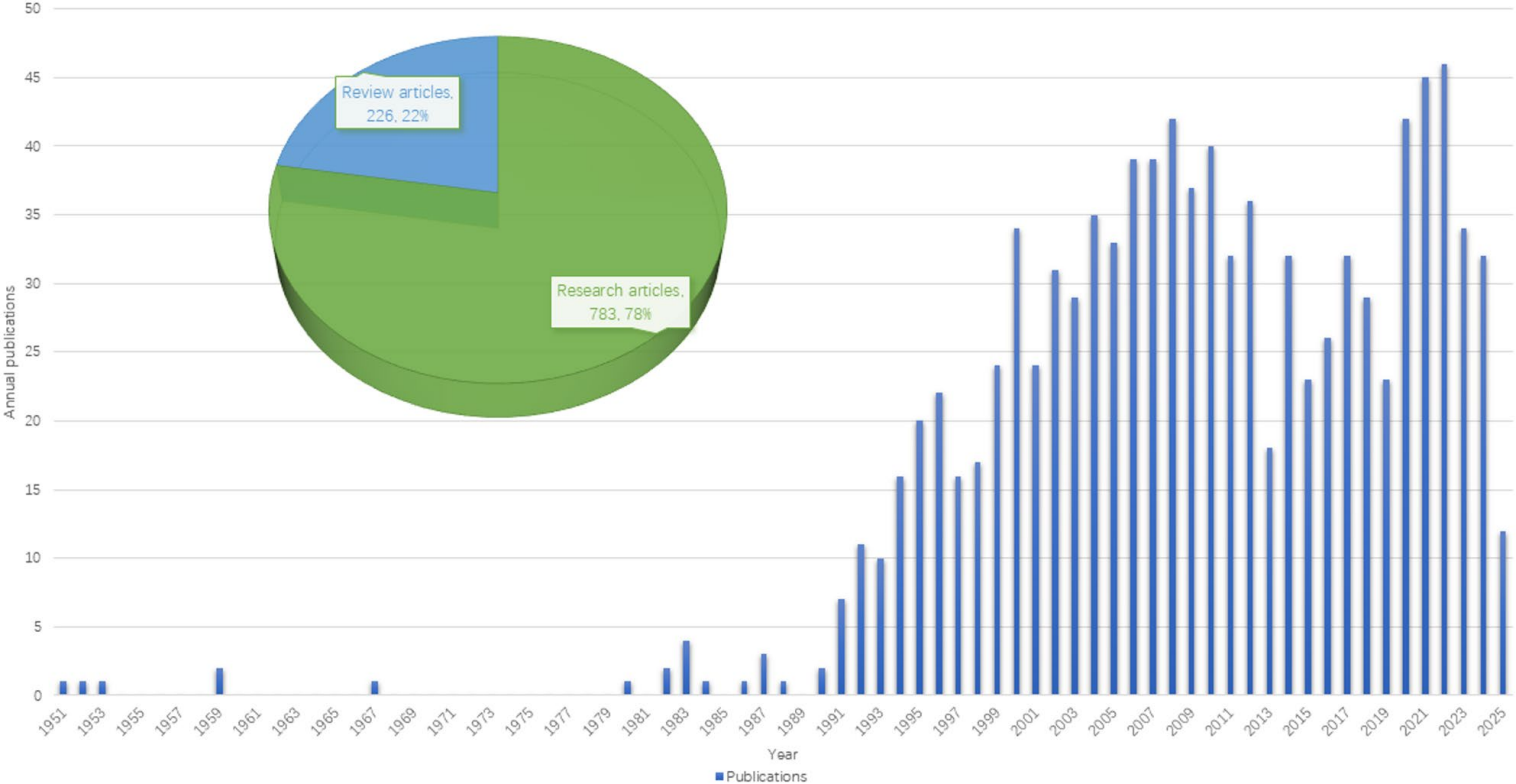
Publication output and trends of estrogen in RA from 1951 to 2025

A total of 1,009 literatures were included in this study. The annual output of this field is shown in Fig. 2. The dataset comprised 783 original research articles and 226 review articles, with the earliest publication dating back to 1951. After 1982, the number of publications in this field began to show an overall growth trend and reached a peak around 2022. From 1951 to 2025, the Annual Growth Rate of publications in this field was 3.41%. Citation frequency serves as a crucial metric for assessing scholarly influence. High citation rates highlight a study’s academic importance and impact on future research. Within this research domain, there have been a total of 59,006 citations, averaging 58.48 citations per paper.

### Analysis of countries/regions

**Fig. 3:**
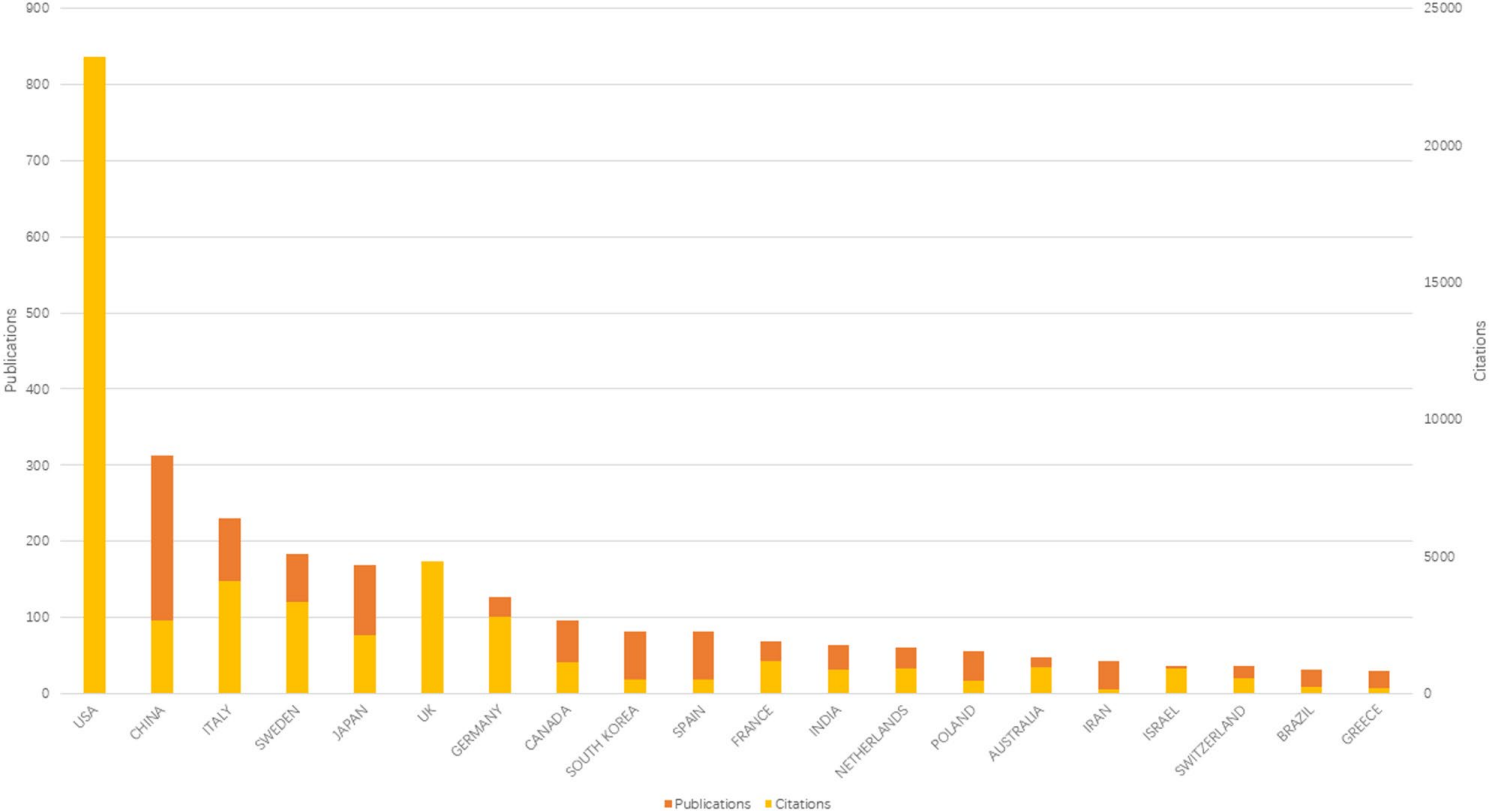
The top 20 countries/regions in terms of publication output and their citations (including all authors)

**Fig. 4:**
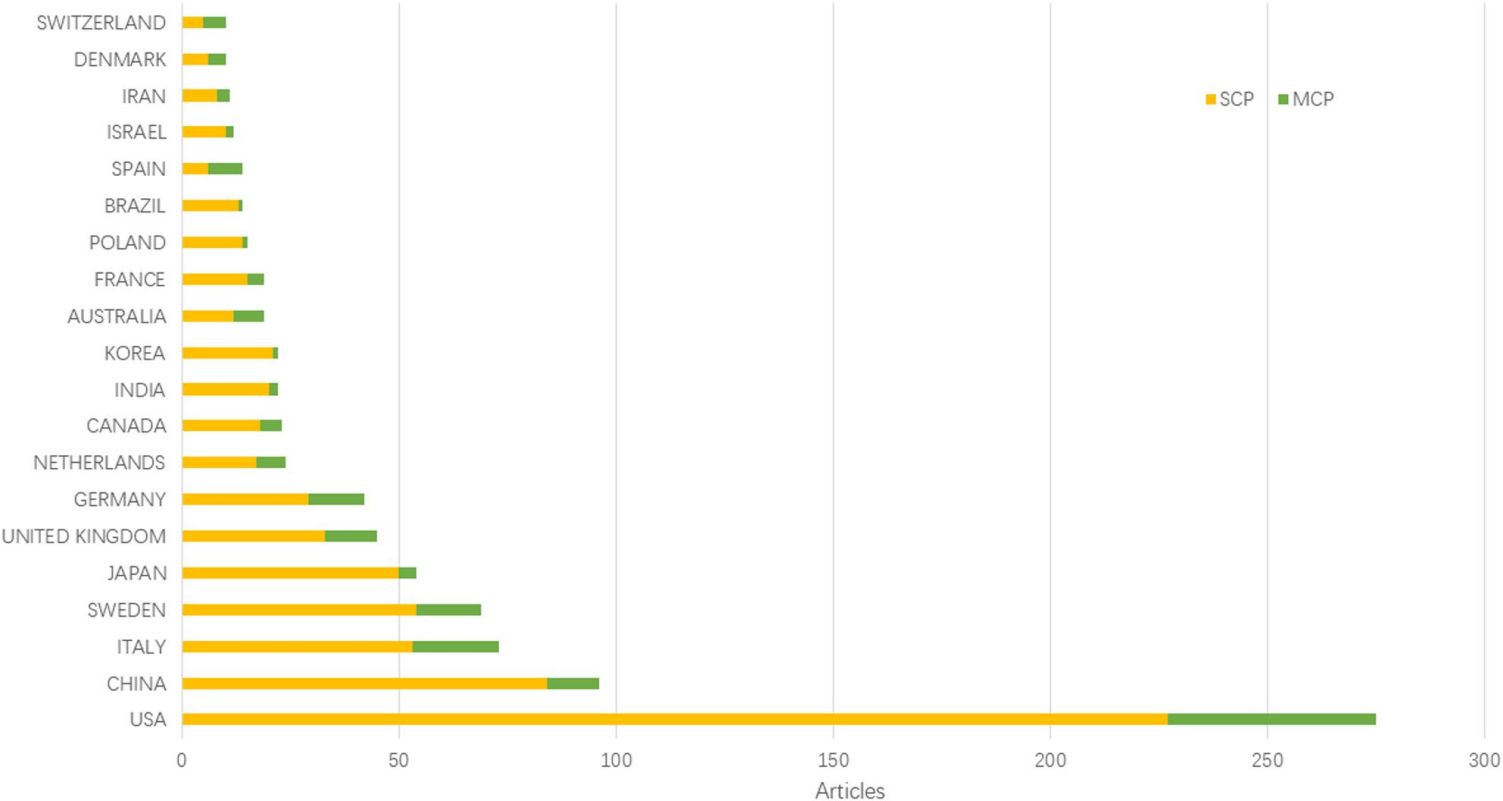
The top 20 countries/regions in terms of publication output (corresponding authors only), SCP, Single Country Publications, MCP, Multiple Country Publications

These 1,009 publications are the result of the efforts of scholars from 60 countries and regions. We use the Bibliometrix software package to count the output of countries/regions. It is divided into two modes. One mode is to include all authors (Fig. 3), and the other is to consider only the corresponding author (Fig. 4). The results show that the United States, China and Italy produce the most publications. The countries with the highest citations are the United States, the United Kingdom and Italy. Although China’s publication output is second only to that of the United States, in terms of global citations, it ranks only eighth. Therefore, China should conduct more in-depth research in this field and enhance its international influence. For instance, although the United Kingdom and Italy are not the countries with the largest output, their citations are still considerable, indicating that their research has a significant impact on the development of this field.

**Fig. 5:**
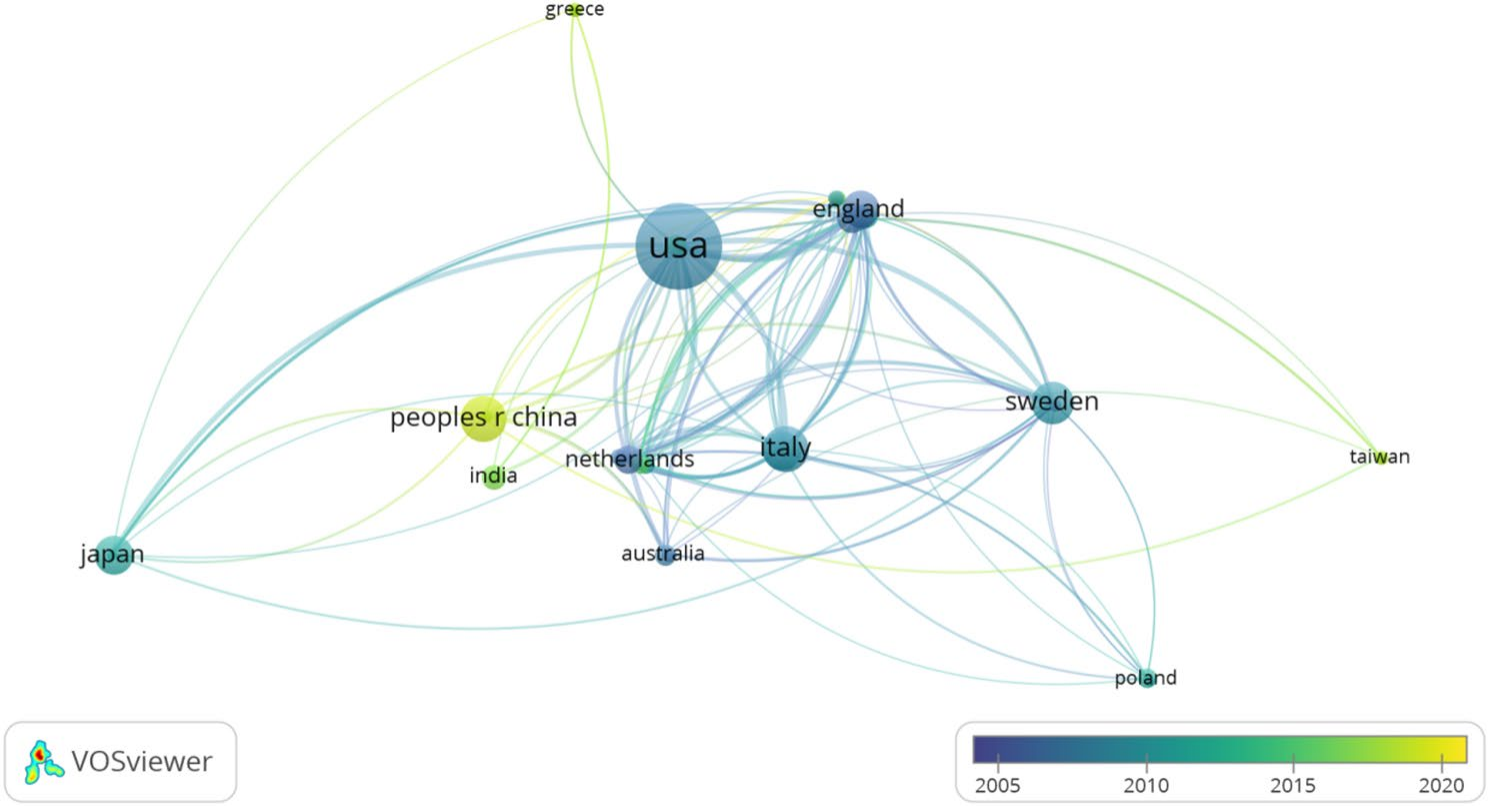
The cooperation network among countries

The cooperation among countries/regions is shown in Figs. 4 and 5. Figure 4 delineates Multiple National Publications (MCP) as those articles co-authored by researchers from different countries or regions, whereas Single National Publications (SCP) are those authored entirely by individuals from the same country or region. It is evident that, in most countries or regions, academic research predominantly occurs domestically, although there exists a foundational level of international cooperation. Among the top ten countries or regions, considering only the corresponding authors, MCP constitutes approximately one-fifth of the publications. Furthermore, we have visualized the international collaboration network using Vosviewer, as depicted in Fig. 5. International collaboration predominantly involves countries and regions that are actively engaged in research within this domain. In the early stage, it was mainly centered on the United States and cooperated relatively closely with the United Kingdom and Italy. In the later stage, China was the core, and cooperation with the United States and Australia was relatively close.

### Analysis of affiliations

**Fig. 6:**
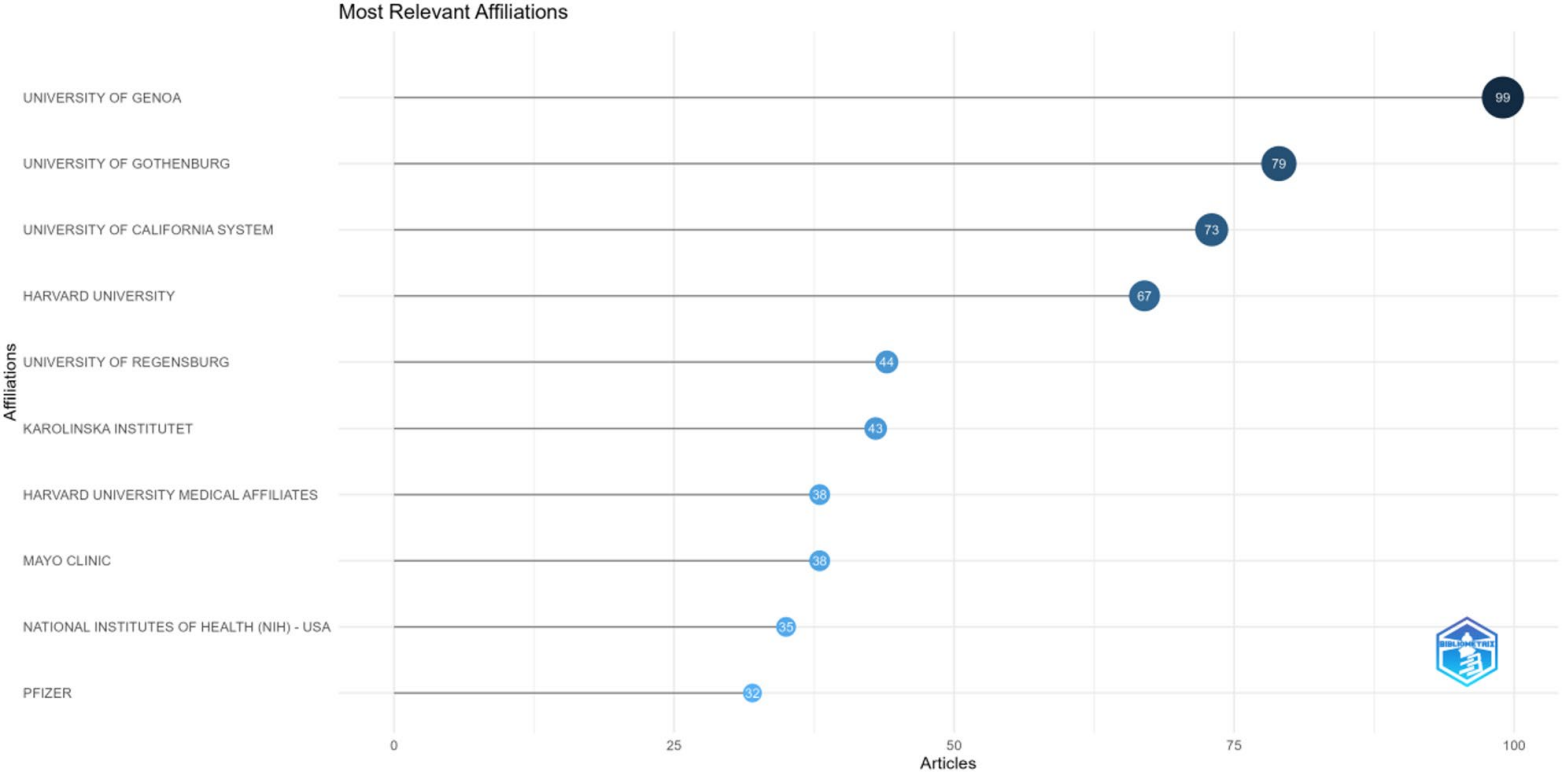
The top 10 organizations by publication output

**Fig. 7:**
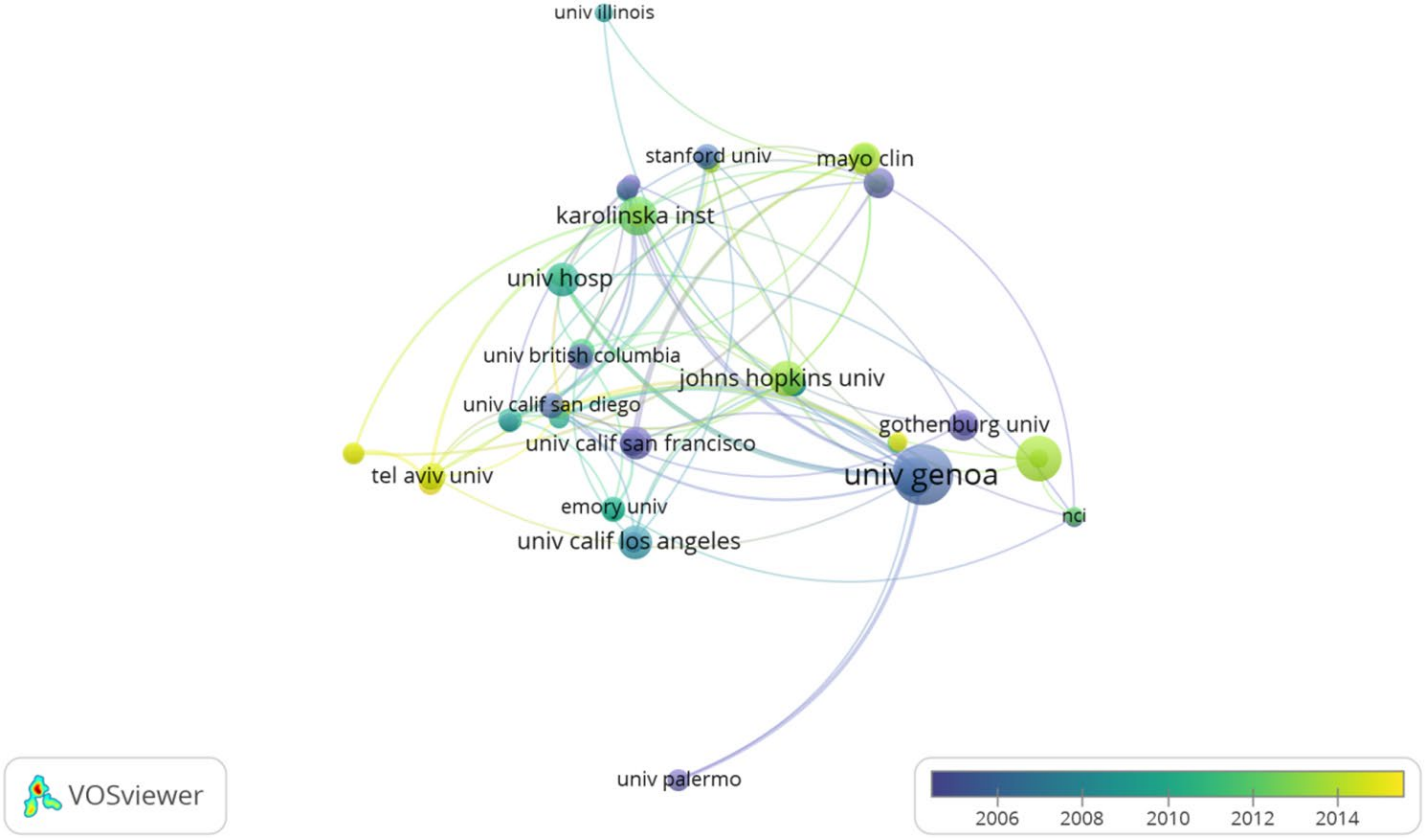
The cooperation network among affiliations

A total of 1,101 affiliations worldwide have conducted research on the field of estrogen related to RA. The top 10 affiliations in terms of publication output in this research field are shown in Fig. 6. The University of Genoa is the university that publishes the largest number of papers, followed by the University of Gothenburg, the University of California System, and Harvard University. All these four universities have produced more than 50 publications. However, approximately four fifths of the institutions have fewer than five articles. It can be seen from the inter-institutional cooperation network diagram (Fig. 7) that the main inter-agency collaborations are concentrated at the University of Genoa, Johns Hopkins University, the University Hospital of Bern, Karolinska Institutet, and the University of California, San Francisco.

### Analysis of authors

**Fig. 8:**
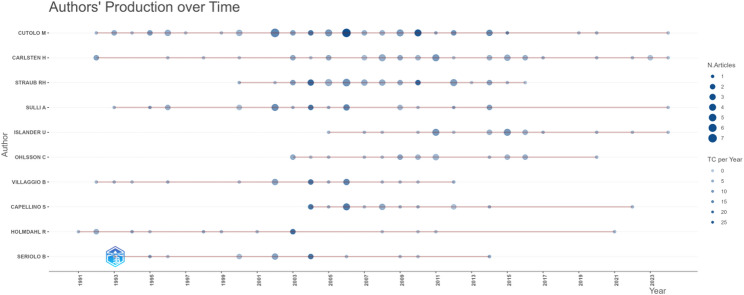
Authors’ production over time

Prolific authors shape research trends and drive advancements. A bibliometric analysis shows that 4,472 scholars worldwide have published on estrogen and RA research. Among them, CUTOLO M leads with 58 papers, accounting for 5.7% of the total, and also ranks first in citations, having an overwhelming advantage. Secondly, there are CARLSTEN H (38 articles) and STRAUB RH (35 articles). Notably, there is no significant variance in the publication output among authors ranked towards the lower end of the top ten (refer to Table 1). Utilizing data on the year of publication, annual publication count, and total citation metrics, Bibliometrix generated the ‘Authors’ Production Over Time’ chart (Fig. 8). As shown in the figure, CUTOLO M and CARLSTEN H have been researching in this field for more than 20 years and are still active, as indicated by their recent publication years.

**Table 1:** The top 10 authors in terms of publication output and the corresponding citations

NO.	Author	Country	Affiliation	Articles	Citations
1	CUTOLO M	Italy	University of Genoa	58	646
2	CARLSTEN H	Sweden	University of Gothenburg	38	272
3	STRAUB RH	Germany	University of Regensburg	35	363
4	SULLI A	Italy	University of Genoa	24	267
5	ISLANDER U	Sweden	University of Gothenburg	21	120
6	OHLSSON C	Sweden	University of Gothenburg	19	162
7	VILLAGGIO B	Italy	University of Genoa	18	267
8	CAPELLINO S	USA	Johns Hopkins University	17	190
9	HOLMDAHL R	Sweden	Lund University	14	118
10	SERIOLO B	Italy	University of Genoa	14	160

### Analysis of journals

**Fig. 9:**
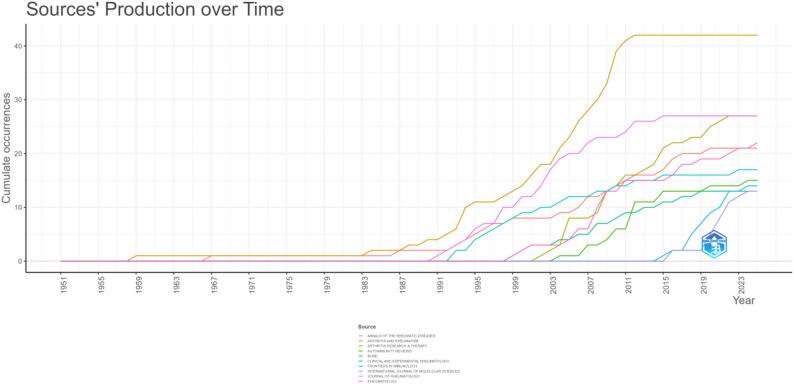
Journals’ production over time

In total, 451 journals have disseminated research articles in the fields of estrogen and RA. Table 2 lists the ten journals with the highest publication frequency in this area. Collectively, the top ten journals account for 211 papers, representing 20.9% of the total publications. The most published articles in this field are from Arthritis and Rheumatology, followed by Arthritis Research and Therapy and the Journal of Rheumatology. Among them, Arthritis and Rheumatology have the most frequently cited ones, which have a gap advantage. After 2003, eight of the top 10 journals began to pay attention to this field (Fig. 9). Around 2007, Arthritis and Rheumatology began to show a gap advantage. By around 2016, all the top 10 journals had started to focus on this field.

**Table 2:** The top 10 journals in terms of publication output and the corresponding citations

NO.	Journal	IF	Articles	Citations
1	Arthritis and Rheumatology	11.4	42	1839
2	Arthritis Research and Therapy	4.4	27	627
3	Journal of Rheumatology	3.6	27	1614
4	Rheumatology	4.7	22	501
5	‌Annals of the Rheumatic Diseases	20.3	21	1715
6	Clinical and Experimental Rheumatology	3.4	17	473
7	Autoimmunity Reviews	9.2	15	356
8	Frontiers in Immunology	5.7	14	326
9	Bone	3.5	13	503
10	International Journal of Molecular Sciences	4.9	13	196

### Analysis of high-cited articles

In bibliometrics, two key concepts are distinguished: “Global Citation Score (GCS)” and “Local Citation Score (LCS).” GCS denotes the total number of citations an article receives within the WOSCC, whereas LCS pertains to the number of citations within a specific collection. LCS is indicative of an article’s impact within the domain of estrogen research related to RA. Table 3 presents the top 10 articles with the highest local citation scores. “CASTAGNETTA LA, 2003, J RHEUMATOL” is notably influential due to its high LCS and relatively high GCS. “CUTOLO M, 1995, CLIN EXP RHEUMATOL” has a large number of GCS, and LCS also ranks fourth, indicating that it also has a considerable influence in this field.

**Table 3:** The top 10 highly local cited articles

NO.	Document	Title	DOI	LCS	GCS
1	CASTAGNETTA LA, 2003, J RHEUMATOL	Increased estrogen formation and estrogen to androgen ratio in the synovial fluid of patients with rheumatoid arthritis	https://www.jrheum.org/content/30/12/2597.long	65	129
2	HALL GM, 1994, ANN RHEUM DIS	A randomised controlled trial of the effect of hormone replacement therapy on disease activity in postmenopausal rheumatoid arthritis	10.1136/ard.53.2.112	52	99
3	D’ELIA HF, 2003, J RHEUMATOL	Influence of hormone replacement therapy on disease progression and bone mineral density in rheumatoid arthritis	https://www.jrheum.org/content/30/7/1456.long	46	93
4	CUTOLO M, 1995, CLIN EXP RHEUMATOL	Estrogens, the immune response and autoimmunity	PMID:7,656,468	43	237
5	CUTOLO M, 1993, ARTHRITIS RHEUM	Presence of estrogen-binding sites on macrophage-like synoviocytes and CD8+, CD29+, CD45RO + T lymphocytes in normal and rheumatoid synovium	10.1002/art.1780360809	42	95
6	HALL GM, 1994, ARTHRITIS RHEUM	Effect of hormone replacement therapy on bone mass in rheumatoid arthritis patients treated with and without steroids	10.1002/art.1780371014	33	210
7	VANDENBRINK HR, 1993, ANN RHEUM DIS	Adjuvant oestrogen therapy does not improve disease activity in postmenopausal patients with rheumatoid arthritis	10.1136/ard.52.12.862	31	48
8	DORAN MF, 2004, J RHEUMATOL	The effect of oral contraceptives and estrogen replacement therapy on the risk of rheumatoid arthritis: a population based study	https://www.jrheum.org/content/31/2/207.long	31	100
9	CUTOLO M, 1996, J CLIN ENDOCR METAB	Androgen and estrogen receptors are present in primary cultures of human synovial macrophages	10.1210/jcem.81.2.8636310	30	114
10	CUTOLO M, 2002, ANN NY ACAD SCI	Androgens and estrogens modulate the immune and inflammatory responses in rheumatoid arthritis	10.1111/j.1749-6632.2002.tb04210.x	30	143

### Analysis of keywords

**Fig. 10:**
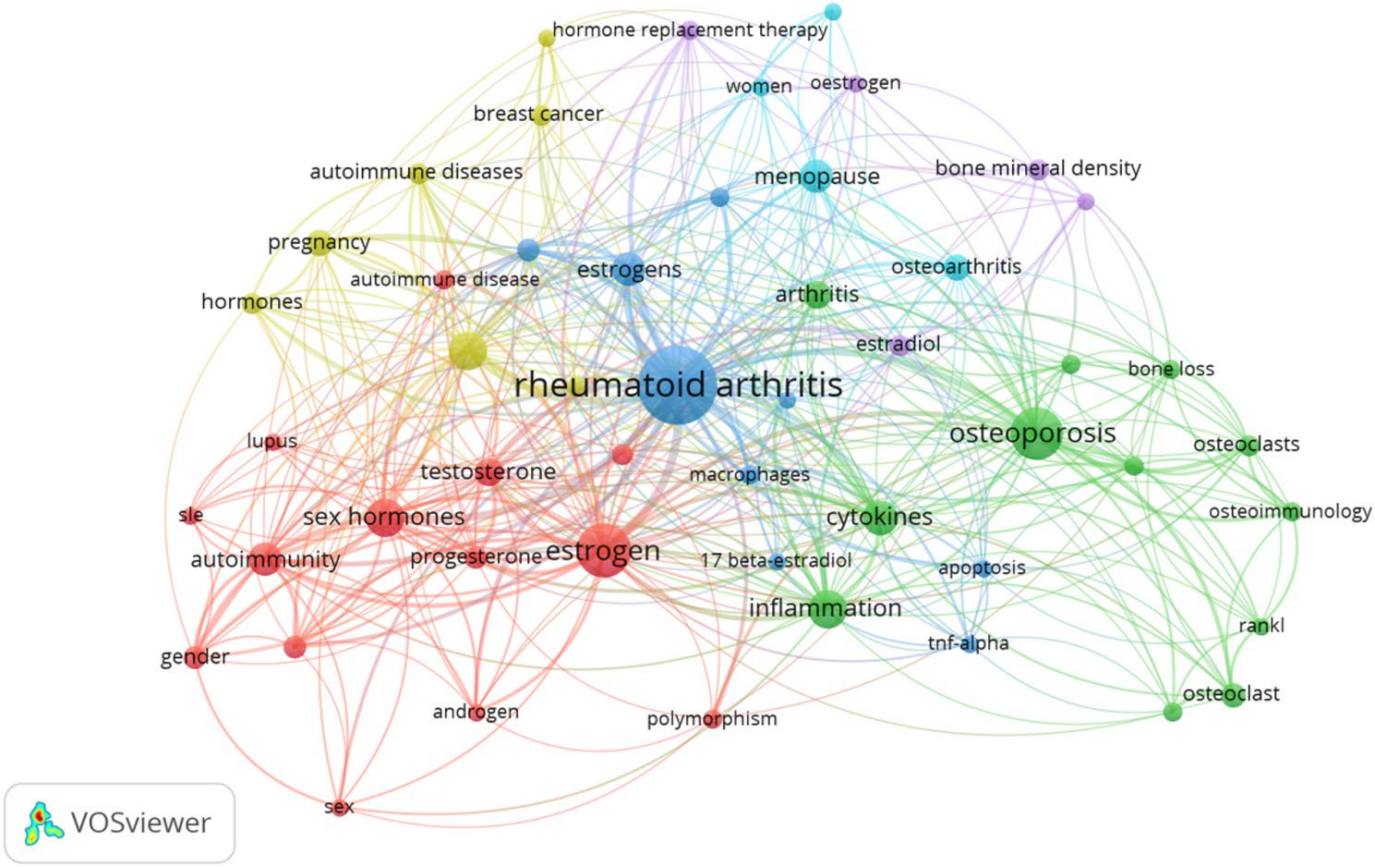
Graph of the keyword co-occurrence network based on clustering

Key words can effectively reflect current trends and cutting-edge research fields. We selected the keywords that co-appeared more than 10 times through VOSviewer, totaling 51 keywords. The top 10 with the highest occurrence frequency are: rheumatoid arthritis, estrogen, systemic lupus erythematosus, osteoporosis, estrogens, inflammation, cytokines, autoimmunity, testosterone, and androgens. These key words form six clusters, as shown in Fig. 10.

The first cluster is red and consists of 14 key words. The key words mainly include “estrogen”, “sex hormones”, “autoimmune”, “androgen”, “progesterone”,“gender”, etc. This also leads to the starting point of the initial research in this field. The gender differences in autoimmune diseases are also an unavoidable topic in the study of estrogen and RA. The second cluster is green and consists of 12 key words. The key words mainly include “osteoporosis”, “inflammation”, “cytokines”, “arthritis”, etc., indicating that this field focuses on the mechanism research of inflammation and cytokines. The third cluster is blue and consists of 9 key words. The key words mainly include “rheumatoid arthritis”, “estrogens”, “androgens”, “glucocorticoids”, “macrophages”, “apoptosis”, etc. This clustering is a common concern in this field, involving some content at the level of the mechanism of action. The fourth cluster is yellow and consists of 7 key words including “pregnancy”, “hormones”, “immune system”, “autoimmune diseases”, etc. It can be seen that this cluster mainly mentions that sex hormones primarily affect autoimmune diseases and may influence pregnancy through certain immune mechanisms. The fifth cluster is purple and consists of 5 key words, such as “estradiol”, “hormone replacement therapy”, “oestrogen”, etc. It can be seen that this cluster mainly reflects some explorations by researchers on regulating estradiol and thereby influencing the condition of RA. The sixth cluster is cyan and consists of 4 keywords, such as “menopause”, “risk factors”, “women”, etc. This cluster mainly reflects the possible impact of estrogen on RA patients in specific age groups.

**Fig. 11:**
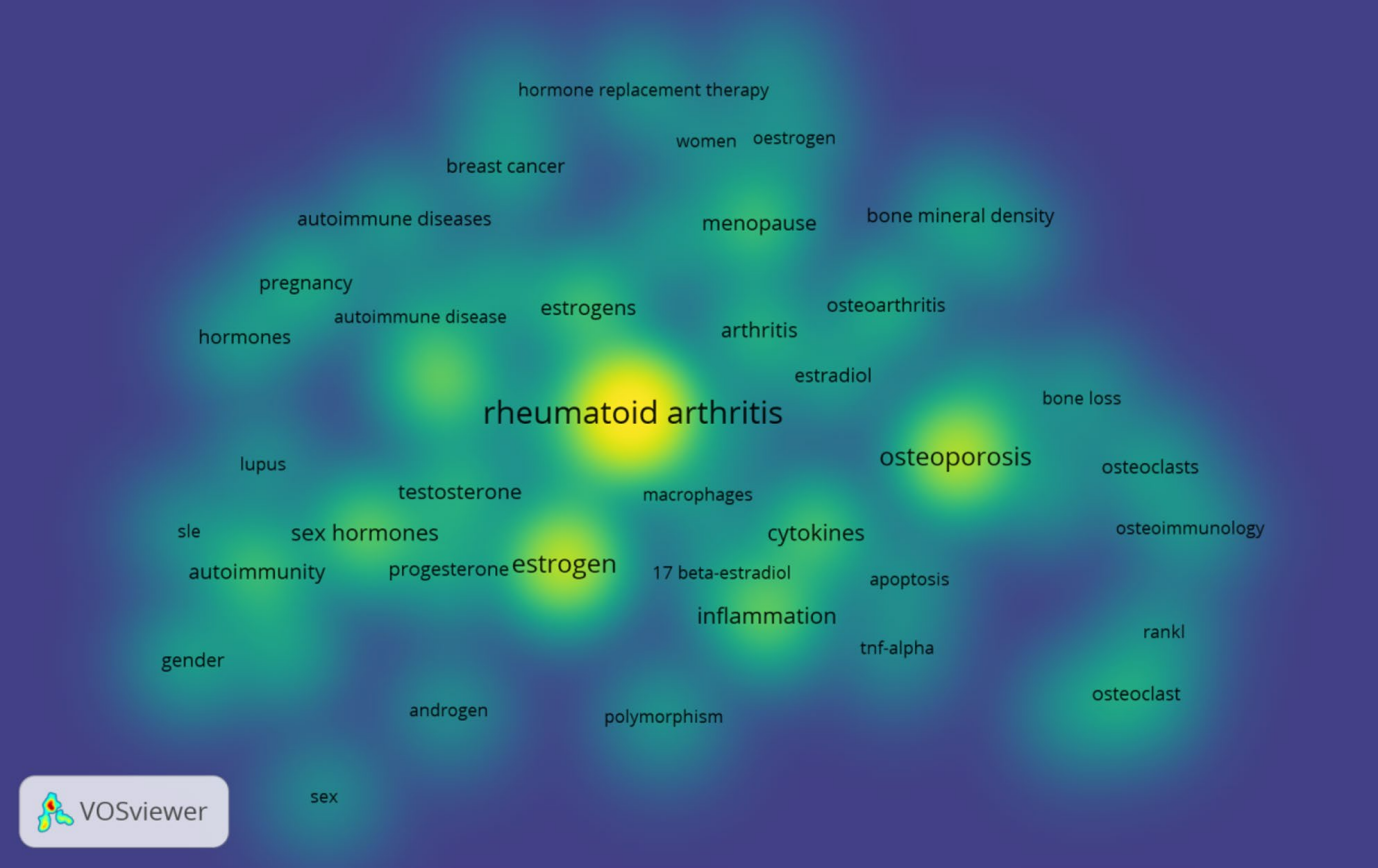
Keyword co-occurrence density map

Figure 11 shows the co-occurrence density map of keywords. The research content of these articles consistently centers around “rheumatoid arthritis” and “estrogen”. Hot keywords include “osteoporosis”, “inflammation”, “cytokines”, “sex hormones”, “macrophages”, “autoimmune”, “menopause”, etc. Analysis of these prevalent keywords reveals critical insights; for example, RA is characterized as an autoimmune disease, and much of the research on estrogen’s role in RA focuses on autoimmune and inflammatory responses. Meanwhile, estrogen runs through the life of women with RA. Menopause is a period of high incidence of RA and also a key focus of research in this field.

### Topic trends

**Fig. 12:**
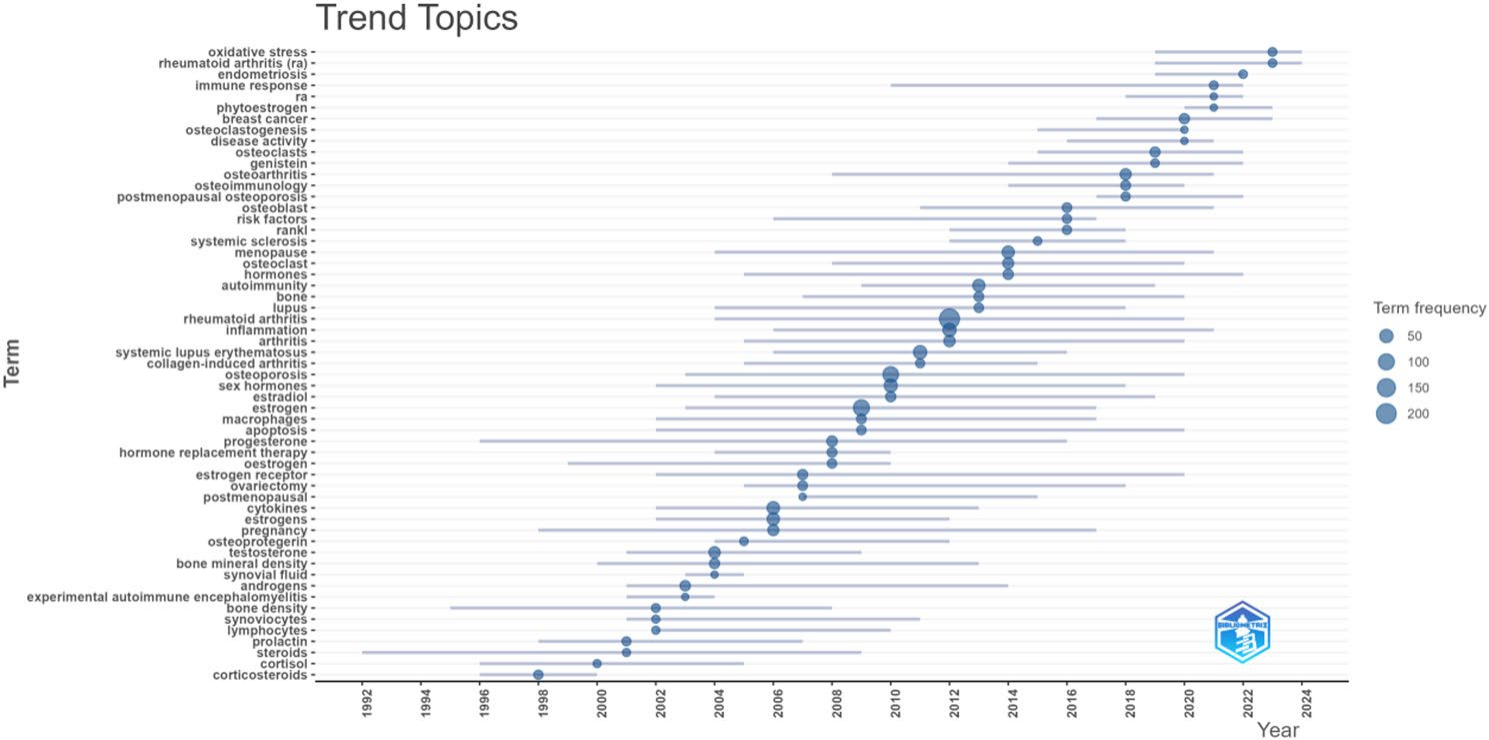
The topic trends in the field of estrogen in RA

The topic trend analysis was conducted using Bibliometrix to understand the research trends. As illustrated in Fig. 12, the initial stage of research output was minimal. Until the year 2000, the continuity of high-frequency keywords was lacking. The high-frequency keywords related to its role in 2006 were “postmenopause”, “cytokines”, “pregnancy”, etc., indicating that many studies focused on exploring the mechanisms of estrogen’s involvement in RA and the influence of estrogen on RA patients at specific ages. The high-frequency key words in the literature from 2009 to 2012 included “macrophages”, “apoptosis”, “inflammation”, “arthritis”, “osteoarthritis”, “systemic lupus erythematosus”, etc. Initially, discussions included autoimmune diseases analogous to RA. These diseases exhibit a significant relationship with immune function and estrogen. Certain pathogenic processes and mechanisms of action share common features, which can offer novel insights for research on estrogen’s role in RA. Secondly, some studies on cellular functions were mentioned, indicating that it is possible that estrogen is involved in the entire process of RA through related cellular functions. Since 2020, there has been an emergence of high-frequency keywords such as “disease activity”, “immune response”, “oxidative stress”, and “endometriosis”. This trend highlights a recent research focus on elucidating the mechanisms of estrogen’s involvement in RA and the shared mechanisms of related diseases.

### Thematic map

**Fig. 13:**
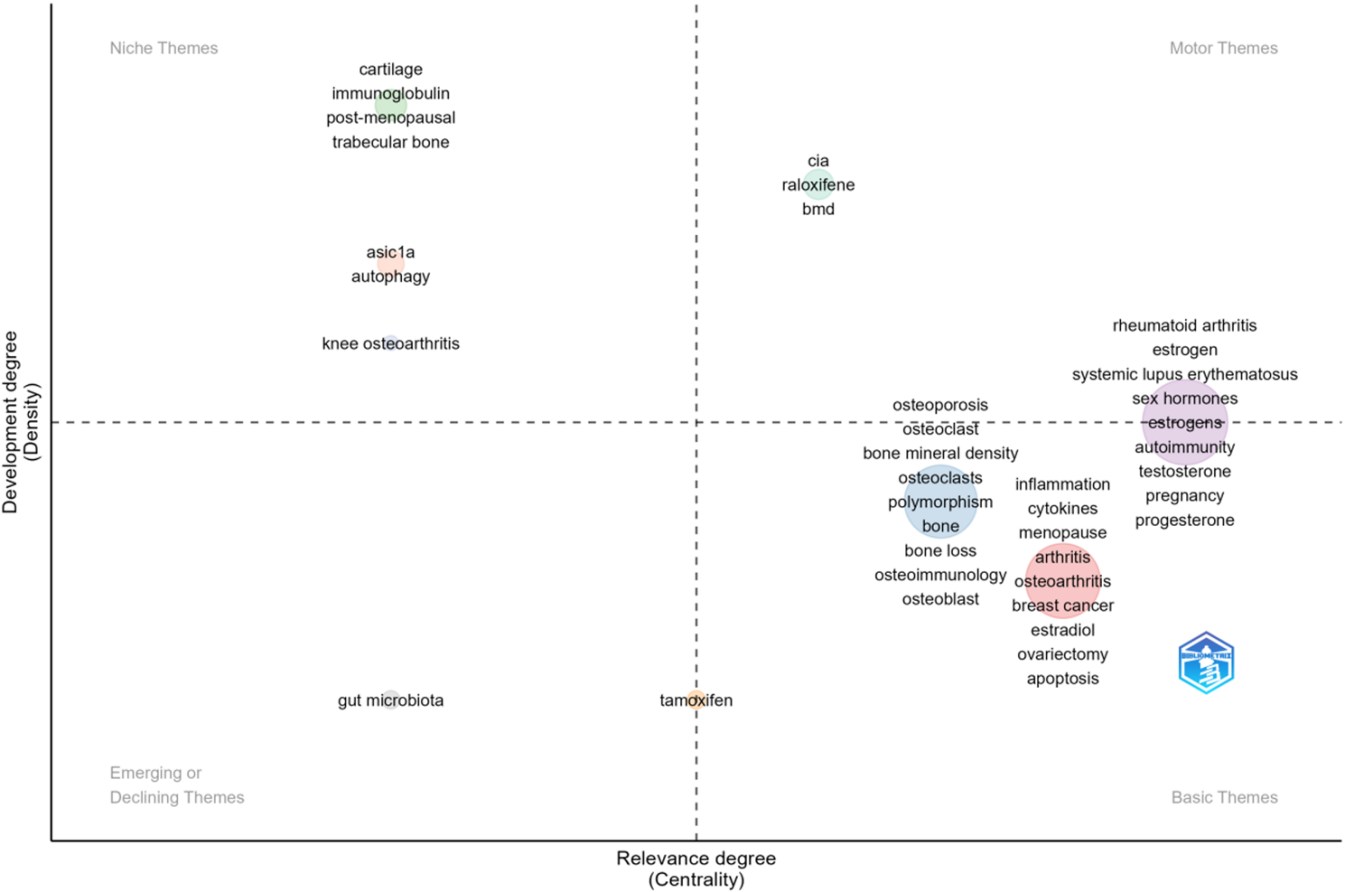
The thematic map of the field of estrogen in RA

The keyword co-occurrence network outlines the main research themes in this field, but identifying future directions is difficult. To assist, the ‘Thematic Map’ module of Bibliometrix was used. In this map, the horizontal axis shows centrality (topic–field association), and the vertical axis shows density (theme development). The map has four quadrants (Fig. 13): the first quadrant (motor theme) includes highly significant and well-developed themes such as “rheumatoid arthritis”, “estrogen”, “systemic lupus erythematosus”, “sex hormones”, “autoimmunity”, and “collagen-induced arthritis (CIA)”, indicating the close connection between estrogen, RA, and the immune system, which is the cornerstone of research in this domain. The second quadrant contains niche themes that are well-developed but less relevant to the field. It mainly includes the key words “cartilage”, “immunoglobulin”, “acid-sensing ion channel 1a (ASIC1a)”, “autophagy”, “knee osteoarthritis”, etc. Among them, the development degrees of “ASIC1a”, “autophagy” and “knee osteoarthritis” were relatively moderate, and the centralization degree was relatively low. While “cartilage” and “immunoglobulin” have developed to a greater extent, they are lacking in centrality. Conversely, themes in the third quadrant, categorized as emerging or declining, are not fully developed; they are either newly emerging or on the verge of obsolescence. Figure 13 highlights “gut microbiota” as the central keyword within this quadrant. Some exploratory studies on intestinal microbiota in this field can be conducted, but it is possible that there may not be good results. The theme in the fourth quadrant (the basic theme) is important in this field, but it is not perfect. One of the topics is the key words such as “inflammation”, “cytokines”, “menopause”, “arthritis”, etc., involving the mechanism and age research of estrogen’s involvement in RA. Another topic has developed well, mainly related to osteoporosis research, such as “osteoporosis”, “osteoclast"and “bone mineral density”.

## Discussion

We conducted a bibliometric analysis of 1,009 literatures on estrogen and RA published in WOSCC from 1900 to 2025 using the bibliometric software VOSviewer and Bibliometricx. The results show that the scientific output in this field as a whole shows an increasing trend. The United States and China are the leading contributors to research in this area. However, analysis of institutions, authors, and highly cited articles shows that Italy also holds a significant position, as the highest-output institutions, most productive authors, and highly cited articles all come from Italy. At the same time, it is also reflected in certain international cooperation. Among the top ten high-impact journals, “Arthritis and Rheumatology” boasts the highest citation count, while the “Annals of the Rheumatic Diseases” possesses the highest impact factor. Through a co-occurrence analysis of keywords, we have identified historical research hotspots in this field, primarily focusing on the mechanisms and applications of estrogen’s role in RA, as well as its effects on RA patients of specific age groups.

Estrogen exerts its biological effects primarily through two nuclear receptors (ERα and ERβ) and a membrane-associated G protein-coupled estrogen receptor. Notably, ERα and ERβ exhibit distinct expression patterns across various immune cell populations and tissues. The functional outcome of estrogen signaling is critically dependent on the relative abundance of these receptor subtypes. In vitro studies have shown that macrophages in RA synovial tissue treated with 17β-estradiol (10⁻⁹ M) for 24 h can enhance the expression of Fas ligand (FAS-L), thereby regulating the process of cell apoptosis [13, 14]. Furthermore, existing studies have demonstrated that 17β-estradiol treatment of RA synovial cells can significantly reduce the elevated levels of transforming growth factor β-activated kinase 1 (TAK1) in RA fibroblast-like synoviocytes (RA-FLS) [15]. In addition, the estrogen-mediated apoptotic effect seems to antagonize hydrogen peroxide-induced programmed cell death, while enhancing the secretion of matrix metalloproteinase-3 stimulated by tumor necrosis factor-α (TNF-α) and increasing the transcriptional activity of CC motif chemokine ligand 13. Moreover, estrogen can also promote the recruitment of inflammatory cells into synovial fibroblast tissues by activating the extracellular signal-regulated kinase 1/2 (ERK1/2) cascade [16]. These processes jointly promote the occurrence and development of RA. The findings from these in vitro studies underscore the intricate molecular mechanisms through which estrogen influences joint tissue activity in RA. Three distinct strains of mice susceptible to CIA were administered 17β-estradiol sustained-release microspheres, resulting in a significant inhibition of arthritis. The therapeutic intervention significantly suppressed T cell-derived interferon (IFN)-γ and interleukin (IL)−10 secretion, while concurrently diminishing granulocyte-macrophage colony-stimulating factor (GM-CSF) production in lymph node-derived immune cells [17]. Ganesan and colleagues investigated the anti-inflammatory effects of estrogen administration in a CIA rat model through comprehensive evaluation of multiple parameters. Their study systematically examined therapeutic outcomes by measuring: (1) paw edema volume as a clinical indicator of disease progression, (2) radiographic changes in joint architecture, (3) histological alterations in synovial tissues, (4) serum concentrations of inflammatory cytokines, (5) biomarkers of bone remodeling, (6) quantification of prostaglandin E2 (PGE2) as a nociceptive mediator, and (7) immunological reactivity to type II collagen. This multifaceted approach provided robust evidence for the therapeutic potential of estrogen in RA [18]. Estrogen is likely a significant factor affecting the changes in the Th1/Th2 and Th17/Treg cell balance observed in pregnant and E2-treated mice [19–21]. In RA patients, regardless of gender, there is a trend toward lower androgen levels and higher estrogen levels in their synovial fluid [22, 23]. The observed effect likely stems from increased concentrations of pro-inflammatory cytokines, including TNF-α, IL-6, and IL-1, present in the synovial tissue of RA patients. These signaling molecules enhance the enzymatic activity of aromatase in extra-gonadal tissues, thereby promoting the metabolic conversion of androgen precursors into estrogenic compounds through the aromatase pathway [24]. Although keyword co-occurrence analysis did not definitively highlight the involvement of microRNAs (miRNAs) in this context, existing research indicates that miRNAs play a crucial role in the pathogenesis and progression of RA [25]. Estrogen is capable of binding to and activating ERα, which subsequently alters the miRNA profile in RA. This alteration enhances the production of key proteins involved in miRNA biogenesis, including Drosha, Dicer, and DGCR8 [26]. Furthermore, keyword co-occurrence analysis suggests a significant association between menopause and this domain. In particular, early menopause is identified as a risk factor for the development of RA, with women experiencing menopause before the age of 45 exhibiting a higher risk compared to those undergoing menopause later [27–29]. Consequently, early menopause results in a reduced duration of estrogen exposure for bone tissue, thereby disrupting the balance between bone formation and resorption and ultimately contributing to bone loss. Estrogen exerts a protective effect on RA [30]. Although hormone replacement therapy (HRT) shows no significant association with the incidence of RA [31], substantial evidence indicates its therapeutic benefits in RA management. Clinical studies have revealed that HRT administration can effectively alleviate clinical manifestations, decelerate pathological progression, mitigate articular destruction and inflammatory responses, promote skeletal mineralization, and optimize subjective health evaluations among affected individuals [32–35]. Notably, HRT also diminishes the risk of anti-citrullinated peptide antibody (ACPA) positivity associated with RA-high-risk HLA alleles [33]. Estrogen deficiency promotes T cell proliferation and extends the lifespan of active T cells via IFN-γ, contributing to bone loss [36]. The bone marrow microenvironment of postmenopausal women demonstrates elevated populations of CD3^+^ T lymphocytes and B lymphocytes that express receptor activator of nuclear factor kappa-B ligand (RANKL). This estrogen deficiency-mediated immunomodulation potentially contributes to the development of osteoporosis in the postmenopausal state [37]. In murine models genetically susceptible to rheumatoid arthritis, estrogen depletion potentially induces the activation of RANKL-expressing CD4^+^ T lymphocytes, subsequently promoting osteoclastogenesis and subsequent bone resorption [38]. Moreover, estrogen has been shown to mitigate mitochondrial dysfunction triggered by ASIC1a activation, thereby exerting protective effects on articular cartilage integrity in ovariectomized rats with adjuvant-induced arthritis [39].

Figure 13 illustrates that a substantial body of research has established a robust foundation for investigations in this domain. However, at present, the mechanism of estrogen on RA remains one-sided, and the research based on the overall situation and in greater depth is still insufficient. This is consistent with the result of literature collation. First of all, there is a contradiction regarding whether estrogen has a counter-effect on RA, although most studies support the protective effect of estrogen on it. Furthermore, the understanding of the internal mechanism of causality is still insufficient. Despite certain advancements in understanding specific immune cells and inflammatory pathways, further research is warranted. In the future, researchers should take a holistic view and connect the existing studies at present. Meanwhile, they can start from the inflammatory level and pay more attention to its deep-seated mechanisms. This is a broad space for exploration.

The interpretation of our findings should be considered in the context of certain limitations. Firstly, it is completely dependent on the WOSCC database, which may omit important journals, non-English literature or conference papers from certain regions, thereby leading to systematic biases in the results. Second, the inherent time-lag in citation accumulation means that recently published articles might be underrepresented, leading to an underestimation of their impact. Third, our search strategy was deliberately restricted to the core terms “estrogen” and “rheumatoid arthritis” to maximize precision. While this ensured a focused dataset, it may have excluded literature employing synonymous or broader terminology. Future research could employ a more expansive search strategy to further contextualize these findings within the broader scientific landscape. Furthermore, as a bibliometric analysis, this study identifies and maps research trends but cannot elucidate the underlying causal mechanisms or intellectual rationale behind these patterns; such explanations require integration with deeper domain knowledge and qualitative assessment. Finally, inferring content based on keywords may not provide a comprehensive overview. Although keywords can indicate the relevance of the topic, they cannot precisely convey the ideas of the publication and may lead to inaccuracies in bibliometric analysis.

## Conclusion

This article has gained a certain understanding of the interaction between estrogen and RA through bibliometric methods. The volume of publications in this field has demonstrated an overall upward trajectory. A growing number of researchers and institutions worldwide are entering this field, with more academic journals focusing on it. China and the United States are particularly active, with notable contributions from Italy. Recently, the study of estrogen’s inflammatory and immune mechanisms in relation to RA has gained prominence. Although a foundational understanding exists, details remain unclear, necessitating further research to improve our knowledge and to develop new RA prevention and treatment strategies.

## Data Availability

No datasets were generated or analysed during the current study.
